# Predictors of poor outcomes in critically ill patients with hematologic malignancy

**DOI:** 10.1186/2197-425X-3-S1-A159

**Published:** 2015-10-01

**Authors:** M Cornish, MB Butler, M Erdogan, RS Green

**Affiliations:** Dalhousie University, Emergency Medicine, Halifax, Canada; Dalhousie University, Critical Care Medicine, Halifax, Canada; Trauma Nova Scotia, Halifax, Canada

## Introduction

Patients with hematologic malignancy (HM) often require admission to the intensive care unit (ICU) due to organ failure through disease progression or complications of treatment. Although there have been improvements in the prognosis of patients with HM, mortality in this patient population remains high. We sought to describe patient characteristics and determinants of outcomes in a Canadian tertiary care setting.

## Objectives

To identify prognostic variables and outcomes in patients with HM who are admitted to the ICU.

## Methods

We performed a structured chart review of all adult patients (age > 18 years) with HM who were admitted to the ICU of a Canadian tertiary care hospital between 2004 and 2014. Outcome measures included ICU length of stay, prolonged ICU stay (> 10 days), ICU readmission, and mortality (ICU, 30-day, 60-day, and 12-month). A logistic regression model was adjusted for potential confounding variables and fitted to determine factors that were predictive of ICU readmission, prolonged ICU stay, and mortality.

## Results

Overall, 206 patients (mean age 51.3 ± 13.6 years; 60% male) with HM were admitted to the ICU during the study period. The most common types of HM were acute leukemia (34.5%) and lymphoma (33.5%). Less than half (44.7%) of patients had a stem cell transplant prior to admission. The most frequent ICU admission diagnosis was respiratory failure (39.8%), followed by hemodynamic instability (37.9%). The mean ICU stay was 3 ± 6 days, with 14.1% of patients requiring prolonged ICU stay. Readmission to the ICU was necessary for 13.1% of patients. During their ICU course, 71.8% of patients required mechanical ventilation and 56.8% required vasopressor therapy.

Of patients admitted to the ICU, 45.6% died during ICU admission, 30-day mortality was 59.2%, 60-day mortality was 62.6%, and 12-month mortality was 74.3%. After adjustment, factors predictive of mortality in the ICU were the need for mechanical ventilation (adjusted estimate [AE] 3.38; 95% CI 1.37, 8.37; P = 0.008) and need for vasopressor therapy (AE 3.65; 95% CI 1.67, 7.96; P = 0.001). A surgical procedure was associated with increased survival (AE 0.21; 95% CI 0.05, 0.86; P = 0.031). For mortality in survivors of ICU admission, myeloma was the only significant predictor of 30-day mortality (AE 0.21; 95% CI 0.06, 0.7; P = 0.011), 60-day mortality (AE 0.15; 95% CI 0.04, 0.52; P = 0.003), or 12-month mortality (AE 0.15; 95% CI 0.04, 0.52; P = 0.003).

## Conclusions

Over a 10-year period, ICU mortality in patients with HM was 45.6% and 12-month mortality was 74.3%. Necessity for mechanical ventilation and vasopressor therapy were significant predictors of mortality in the ICU, while myeloma was predictive of longer term mortality.

## Grant Acknowledgment

This study was funded by a Clinician Scientist Award from the Faculty of Medicine, Dalhousie University, Halifax, Nova Scotia, Canada.

